# Single-Particle Tracking Reveals Anti-Persistent Subdiffusion in Cell Extracts

**DOI:** 10.3390/e23070892

**Published:** 2021-07-13

**Authors:** Konstantin Speckner, Matthias Weiss

**Affiliations:** Experimental Physics I, University of Bayreuth, Universitätsstr. 30, D-95447 Bayreuth, Germany; konstantin.speckner@uni-bayreuth.de

**Keywords:** anomalous diffusion, random walk, single-particle tracking

## Abstract

Single-particle tracking (SPT) has become a powerful tool to quantify transport phenomena in complex media with unprecedented detail. Based on the reconstruction of individual trajectories, a wealth of informative measures become available for each particle, allowing for a detailed comparison with theoretical predictions. While SPT has been used frequently to explore diffusive transport in artificial fluids and inside living cells, intermediate systems, i.e., biochemically active cell extracts, have been studied only sparsely. Extracts derived from the eggs of the clawfrog *Xenopus laevis*, for example, are known for their ability to support and mimic vital processes of cells, emphasizing the need to explore also the transport phenomena of nano-sized particles in such extracts. Here, we have performed extensive SPT on beads with 20 nm radius in native and chemically treated Xenopus extracts. By analyzing a variety of distinct measures, we show that these beads feature an anti-persistent subdiffusion that is consistent with fractional Brownian motion. Chemical treatments did not grossly alter this finding, suggesting that the high degree of macromolecular crowding in Xenopus extracts equips the fluid with a viscoelastic modulus, hence enforcing particles to perform random walks with a significant anti-persistent memory kernel.

## 1. Introduction

Quantifying transport phenomena in soft and living matter on mesoscopic scales virtually always involves optical microscopy techniques, due to their high spatiotemporal resolution. Presumably, the most informative approach in this context is single-particle tracking (SPT). In SPT experiments, the rapid imaging of a sparse set of (fluorescent) particles, e.g., molecules, beads, quantum dots, or even whole organelles, allows for retrieving individual particle positions over time, eventually providing complete trajectories (see, for example, Refs. [1,2,3] for reviews and [4] for a quantitative comparison of SPT to other techniques). Direct access to particle trajectories facilitates the application of refined analysis approaches [5], with the mean square displacement (MSD) supposedly being the easiest and most familiar measure.

Having SPT data at hand, one can calculate, for example, the time-averaged MSD (TA-MSD), ${\langle r^{2}\left(\tau \right)\rangle }_{t}$, for each trajectory and compare these to their ensemble-average, ${\langle r^{2}\left(\tau \right)\rangle }_{t,E}$. A commonly observed feature is a power-law scaling of both MSDs ${\langle r^{2}\left(\tau \right)\rangle }_{t,E}∼{\langle r^{2}\left(\tau \right)\rangle }_{t}∼\tau^{\alpha }$, with normal Brownian diffusion being indicated by α=1. Scaling exponents α<1 are commonly referred to as ‘subdiffusion’, whereas values 1<α<2 are termed ‘superdiffusion’. Subdiffusion with scaling exponents 0.3<α<0.9 has been observed very frequently, at least on short and intermediate time scales, for tracer particles in complex media, e.g., in equilibrated biomimetic crowded fluids [6,7,8,9,10], in the cytoplasm [6,11,12,13,14,15] and in the nucleoplasm [11,16,17,18] of living cells, as well as on biomembranes [19,20,21,22]; see also [23,24] for extensive reviews.

Cell extracts, which are basically only the cytosol of an ensemble of cells without larger organelle compartments, constitute an intermediate between artificial equilibrated media and nonequilibrium fluids inside living cells. Although extracts support vital processes, such as gene transcription [25] or even the formation of a mitotic spindle [26], diffusional transport in these fluids has so far been explored only sparsely. Biochemically active extracts may be derived from different sources, with the eggs of the clawfrog *Xenopus laevis* supposedly being the most popular: here, unfertilized oocytes are collected from the spawn, cooled down and crushed by centrifugation (see [27] for details). Due to different buoyancies, most membranes and the yolk can be separated from the aqueous cytosol, which eventually is obtained as extract. This extract not only includes all necessary biomolecules at physiological concentrations, but also allows for native interactions of proteins and/or nucleic acids and/or sugars. Due to its amphibian origin, the Xenopus extract provides full functionality, e.g., a dynamic cytoskeleton and even functional spindles, already at temperatures around 20 ${}^{\circ }$C [27]. Given the crowdedness of these fluids and their inherent nonequilibrium background noise due to active proteins, it is unclear how particles explore such a (nearly) living fluid. While an early study, which was focused on the rheology of Xenopus extracts, revealed already that particles with sizes around 1μm move subdiffusively in these fluids [28], a study on smaller particles but also a detailed investigation of the random walk process associated with the observed subdiffusion has been lacking so far.

As a stochastic process, subdiffusion may arise when the accessible space has a fractal geometry [29], e.g., imposed by a sufficiently dense set of immobile obstacles that form a random percolation cluster. In most experimentally relevant cases, however, obstacles are too mobile to induce an obstructed random walk in a fractal environment (see [20] for a discussion). Power-law distributed waiting times between successive steps can also induce subdiffusion [30], yet at the cost of weak ergodicity breaking [31,32], i.e., the scaling of TA-MSDs is that of normal Brownian motion (α=1), whereas a simple ensemble-averaged MSD of many trajectories shows subdiffusive scaling. The difference between both measures, at least in the unconfined case (see [33,34] for results in confined geometries), is related to a successive aging of the system. While such processes were sometimes observed [13,19], most experimental reports are more consistent with fully ergodic random walk processes, albeit often with a marked anti-correlation of successive steps instead of a purely Markovian random walk. For an abstract description of such a subdiffusive mode of motion, fractional Brownian motion (FBM) [35] with a Hurst coefficient H=α/2≤1/2 was used in many cases (see [5,23] for an extensive discussion). In a nutshell, FBM is a Gaussian process with stationary increments whose anti-persistent memory kernel may encode the viscoelastic characteristics of the surrounding medium. Using SPT, experimental data can be tested directly for FBM features not only via MSDs, but also via the power-spectral density (PSD) and correlation functions that report on the memory kernel [5,23].

Here, we show via SPT that beads with 20 nm radius move subdiffusively in native and chemically treated Xenopus extracts. A sublinear scaling of MSDs with an average scaling of 〈α〉≈0.9 is found, accompanied by a significant anti-persistence peak in the velocity autocorrelation function (VACF). The VACF shows excellent agreement with the FBM prediction, and the distribution of step increments is Gaussian, suggesting that the particles perform a subdiffusive random walk of the FBM type. Further support of this notion is given by the PSD and the associated coefficient of variation, both of which also agree very well with the FBM predictions. Chemical treatments of the extract, e.g., depolymerizing the cytoskeleton, do not grossly alter the results, suggesting that the high degree of crowding in Xenopus extracts equips the fluid with a viscoelastic modulus that forces particles to perform FBM-like random walks.

## 2. Materials and Methods

### 2.1. Microscopy and Single-Particle Tracking

Fluorescence images were taken with a customized spinning-disk confocal microscope, consisting of a Leica DMI 6000 microscope stand (Leica Microsystems, Wetzlar, Germany) equipped with a CSU-X1 spinning disk unit (Yokogawa Electric, Tokyo, Japan). Samples were illuminated by a 491/561 nm dual-combined DPSS laser (Cobolt, Stockholm, Sweden), and fluorescence was detected in the range of 500–550 nm or 575–625 nm, respectively. The setup was controlled by a custom written LabView software (National Instruments, Austin, TX, USA). Time series of images were recorded at room temperature (about 19 ${}^{\circ }$C) with a Hamamatsu Orca Flash 4V2.0 sCMOS camera (Hamamatsu Photonics, Hamamatsu City, Japan ), using an HCPL APO 63x/1.4 oil immersion objective (Leica Microsystems). With a 2×2 hardware camera binning, the size of the squared pixels was determined as 112.4 nm.

Rhodamine-labeled microtubules in Xenopus extracts were imaged with an exposure time of 250 ms, using the 561 nm excitation channel. To improve the contrast between microtubules and unbound fluorescent tubulin monomers, images were post-processed in Fiji: the images were filtered with a median filter (radius set to 0.7 pixels), and background fluorescence was removed using a rolling-ball algorithm with a 10-pixel radius (built-in function ‘subtract background’). Subsequently, the colormap *mpl-viridis* was assigned to all fluorescence images.

In our SPT experiments, fluorescent polystyrene microspheres with a diameter of 40 nm (FluoSpheres NeutrAvidin-Labeled, F8771, Thermo Fisher Scientific, Dreieich, Germany) were used. In contrast to carboxylate surface coupling, neutravidin minimizes unspecific interactions with DNA and RNA complexes or negatively charged surfaces. For calibration measurements, 1:100 stock solutions in DNase/RNase-Free Distilled water (Invitrogen) or 1:20 stock solutions (for Xenopus egg extract experiments) were prepared. On average, about 200–400 fluorescent particles were observed in the field of view of the camera sensor ($110\times 70\;\mu m^{2}$). For tracking, 2000 images were recorded with an exposure time of Δt=25 ms per frame, using the 491 nm excitation channel.

Particle positions were detected and linked to trajectories by the ImageJ/FIJI plug-in TrackMate [36]. As an input parameter for TrackMate, the diameter of fluorescent particles was estimated via the intensity profiles of 119 particles embedded in pure glycerol, yielding an average full width half maximum of the point-spread function of 2.9±0.5 pixels. Particle tracking was performed using the Laplacian-of-Gaussian detection algorithm (diameter set to four pixels, threshold set to 50±15 grey values and using sub-pixel localization). No additional filters were applied to the detected spots. Identified particle positions were linked with the simple linear assignment problem tracker adopted from [37]. Here, a maximum linking distance of three pixels was used and frame gaps were not allowed. The minimum trajectory length was set to N≥50 positions and trajectories with a total displacement of less than one pixel were discarded.

Non-assigned detections were cleaned from the time series experiments; particle trajectories were exported as XML and converted to ASCII files for further processing in Matlab (Matlab 2018b, The Mathworks Inc., Natick, MA, USA). All statistical analyses of particle trajectories were performed with custom-written codes in Matlab that were prior checked for proper function by random walk simulation data. In our analyses, particle trajectories were clipped exactly to lengths of N=70 or N=150 time steps for better comparability within the ensemble (i.e., all shorter trajectories were discarded for the analysis). In total, our ensemble (=the number *M* of trajectories of a given length for a given condition) consisted of 1000–3000 trajectories (N=70) and 150–600 (N=150) for Xenopus extract experiments. For varying glycerol water mixtures, 1000–5000 (N=70) and 75–1000 (N=150) trajectories were available. The specific ensemble sizes are given in Tables 1 and 2.

### 2.2. Xenopus Extract Preparation and Modification

Cytostatic factor-arrested (CSF) cytoplasmic extracts were prepared from freshly laid *Xenopus laevis* eggs based on standard protocols [26,27,38]. In brief, eggs in the metaphase stage of meiosis II were collected, dejellied and packed into a centrifugation tube. The packed eggs were crushed and fractioned into three distinctive layers by centrifugation. The mid cytoplasmic layer was carefully isolated and supplemented with 10μg/mL of protease inhibitors, leupeptin, pepstatin and chymostatin (diluted in DMSO), 10μg/mL of cytochalasin D, and ATP regeneration mix (190 mM creatine phosphate, 25 mM adenosine triphosphate, 25 mM ${\mathrm{MgCl}}_{2}$ and 2.5 mM K-EGTA at pH 7.7) that was diluted 1:50 to the extract. Finally, 0.35μL from the 1:20 stock solution of fluorescent particles was added to 20 μL of the extract; optionally, pharmaceuticals for affecting microtubule structures were added. Microtubules were labeled by fluorescent tubulin (TL 331M, Cytoskeleton Inc., Denver, CO, USA) according to the manufacturer’s protocol: 10 mg/mL stock solution of fluorescent tubulin dissolved in general tubulin buffer (80 mM PIPES, 0.5 mM EGTA and 2 mM ${\mathrm{MgCl}}_{2}$ at pH 6.9) with 1 mM GTP that was held on ice and added to Xenopus extracts to a working concentration of 50 μg/mL. The CSF extract was chilled immediately on ice and used within a maximum of two hours for the experiments.

Different chemicals were optionally added to Xenopus extracts to affect the microtubule integrity. Here, attention was paid to dilute the extract as little as possible. Preliminary investigations have shown that a total dilution of Xenopus extracts by up to 10% (due to the addition of beads or drugs) appears unproblematic and does not affect the microtubule structures observed. For all experimental conditions, the total volume added for modifying microtubules was balanced by the addition of distilled water to the otherwise untreated extract.

Nocodazole (Sigma-Aldrich, Munich, Germany) was used to depolymerize microtubules [18]. To this end, a stock solution of 10 mM dissolved in dimethylsulfoxide was diluted to a final concentration of 33.3 μM in Xenopus extract that was kept on ice for 10 min. Afterward, the extract was incubated for 15 min at 25 ${}^{\circ }$C and then transferred to microscopy. Paclitaxel (in the remainder referred to as ‘taxol’, Sigma Aldrich, Germany) was used to stabilize the microtubules. It was added to the extract at a working concentration of 25μM. Non-hydrolizable analogues of ATP (ATPγS, Merck, Darmstadt, Germany) or GTP (GTPγS, Merck, Darmstadt, Germany) were used to affect the turnover of chemical energy in the extract. To this end, ATPγS and GTPγS (stored as stock solutions of 25 mM for ATPγS and 12.5 mM for GTPγS in distilled water) were added to final concentrations of 500μM and 250μM to the Xenopus extracts.

## 3. Results and Discussion

### 3.1. Calibration Experiments in Viscous Media

To arrive at a proper baseline for our SPT experiments, we first tracked fluorescent beads with a radius of R=20 nm in purely viscous media with varying viscosity (see Section 2 for details). In particular, we used here glycerol–water mixtures in the range of 70–90% (per weight) for which the viscosity values are known from the literature. Adding 8% of the bead stock solution to the total volume of these mixtures, viscosities in the range η∈[0.016,0.096]Pa·s were probed by our SPT experiments. For consistency among the trajectories and for better comparison to subsequent experiments in Xenopus cell extracts, we fixed the trajectory length to N=70 or alternatively to N=150, bearing in mind that short trajectories may suffer from statistical fluctuations [39] while longer trajectories might show a bias for picking slower particles [15] that are easier to track without losing them (e.g., due to leaving the focal plane).

Two-dimensional trajectories $r\left(t\right)=r_{1},\ldots ,r_{N}$ acquired at discrete times t=Δt,2Δt,…,NΔt with frame time Δt=25 ms and a total measurement time T=NΔt were first evaluated with their individual TA-MSDs, defined via the following:

(1) $${\langle r^{2}\left(\tau \right)\rangle }_{t}=\frac{1}{N-k}\sum_{j=1}^{N-k}{\left(r_{j+k}-r_{j}\right)}^{2}\;\;,$$

where τ=kΔt denotes the lag time. The resulting TA-MSDs of trajectories were fitted with a simple power-law as follows:

(2) $$\langle r^{2}\left(\tau \right)\rangle =4K\tau^{\alpha }$$

in the range τ∈[0.05,0.3] s by linear regression of log[τ] versus $\log[{\langle r^{2}\left(\tau \right)\rangle }_{t}]$. Therefore, the generalized diffusion coefficient *K* becomes equivalent to the familiar diffusion constant, *D*, for normal Brownian motion (α=1). In this case, the Einstein–Stokes relation predicts the diffusion constant to depend on particle radius *R* and medium viscosity η as follows:

(3) $$D=\frac{k_{B}T}{6\pi \eta R}$$

yielding predictions for diffusion constants in the range $D\in \left[0.11,0.67\right]\;\mu m^{2}/s$ for our SPT experiments in glycerol–water mixtures.

**Figure 1:**
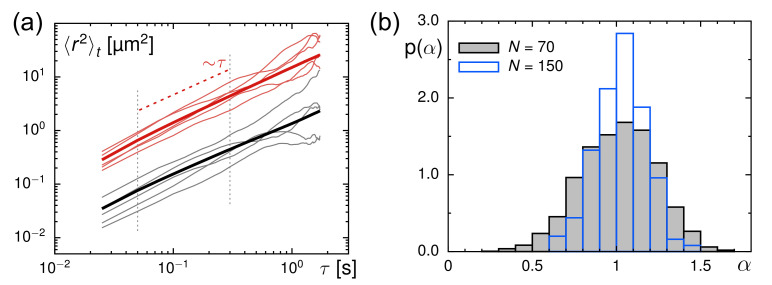
(**a**) Representative TA-MSDs for trajectories with length N=70 (randomly chosen from the ensemble) from experiments in glycerol–water mixtures (red thin lines) and in Xenopus extract (black thin lines), together with the respective ensemble-averaged TA-MSDs (colored thick lines). For better visibility, data for calibration experiments have been shifted upward tenfold. The scaling for normal diffusion (${\langle r^{2}\left(\tau \right)\rangle }_{t}∼\tau$) is indicated by a red dashed line; vertical grey dashed lines indicate the fit region used to analyze individual TA-MSDs. (**b**) The PDF of anomaly exponents, p(α), as obtained from fitting TA-MSDs in glycerol–water mixtures features a mean 〈α〉≈1, irrespective of the trajectory length (black-grey histogram: N=70, blue histogram: N=150).

In line with our expectations for purely viscous fluids, we observed on average normal diffusion, i.e., 〈α〉=1, for all viscosities and trajectory lengths (see summary in Table 1), albeit the individual TA-MSD scaling exponents showing marked fluctuations in the range α∈[0.8,1.2] (see Figure 1 for representative MSDs and the probability density function of scaling exponents, p(α)) due to the limiting statistics in TA-MSDs (see [39] for a detailed discussion). Therefore, the extracted diffusion coefficients *K* also showed marked fluctuations, and, yet again the average overall trajectories revealed a value 〈K〉 that compared favorably to the predicted values of *D* (cf. Table 1). This finding also indicates that particle radii are, on average, near to their declared and expected value, in line with previous findings [4]. Please note the slight but visible bias toward lower values of *K* for longer trajectories, indicating an unwanted bias toward slower particles that could be tracked over longer periods. Moreover, the amount of trajectories available for the analysis clearly increases for increasing viscosity, since slower-moving particles can be tracked easier. Overall, these calibration experiments demonstrate that normal diffusion with the anticipated mobility is found via SPT in purely viscous fluids. Deviations from unity in the (mean) scaling exponent of MSDs can therefore be taken as a clear signature of a significant anomalous diffusion.

### 3.2. Evaluation of Tracer Motion in Native Xenopus Extract

As a next step, we explored the diffusive motion of the same fluorescent particles in Xenopus extracts (see Materials and Methods for details). Given that these extracts are complex and crowded fluids with an active biochemistry, we anticipated considerable differences to the simple glycerol–water mixtures. As before, we restricted ourselves to trajectory lengths N=70 and N=150, and used the TA-MSD and its ensemble average for a first characterization. Representative TA-MSDs are shown together with the ensemble average in Figure 1a.

Next, we fitted all TA-MSDs with Equation (2) to extract the respective scaling exponents, α, and generalized diffusion coefficients, *K*. Here, we tacitly assume that static and dynamic localization errors are negligible for our data; we will confirm this assumption below. Still, to soften any remnant influence of localization errors, especially for retrieving the scaling exponent α, we did not take the first point of TA-MSDs (at τ=Δt) into account for fitting. Fitting was performed as in the calibration measurements.

**Figure 2:**
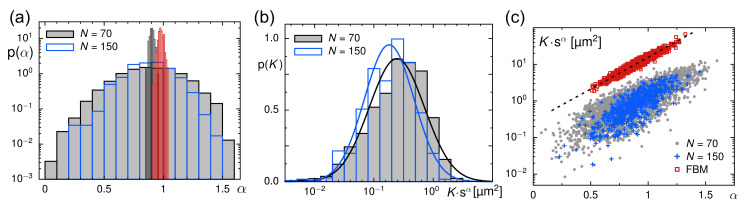
(**a**) The PDF of anomaly exponents, p(α), as obtained from fitting TA-MSDs in the interval τ∈[0.05,0.3] s, features a mean 〈α〉≈0.9, irrespective of the trajectory length (black-grey histogram: N=70, blue histogram: N=150). The considerable width of the PDF may not only reflect statistical fluctuations but is likely to also report on spatially varying material properties of the Xenopus extract. Performing a bootstrapping approach with geometric averaging (black-open histogram) confirms the slightly subdiffusive motion of particles, while an arithmetic averaging (red histogram) overestimates the mean scaling exponent; see also main text for discussion. Please note the logarithmic *y*-axis. (**b**) The PDF of generalized diffusion coefficients, p(K), shown here versus the average area covered in one second, $K\times 1s^{\alpha }$, features an almost lognormal shape (indicated by full lines) for trajectory lengths N=70 (grey/black) and N=150 (blue), with a slight tendency for lower mobilities in longer trajectories. Please see the main text for discussion. (**c**) A scatter plot of trajectory-wise values of α and *K* (blue and grey symbols) highlights a correlation between these two quantities, in good agreement with results on simulated FBM trajectories with a Hurst coefficient H=α/2=0.45 (red symbols). The black dashed line is an empiric guide for the eye. FBM simulation data have been shifted upward fivefold for better visibility.

Evaluating all TA-MSDs yielded probability density functions (PDFs) for the scaling exponent, p(α), and the generalized diffusion coefficient, p(K). Inspecting p(α) (shown in Figure 2a) reveals that the ensemble of trajectories shows, on average, a slight subdiffusive scaling of TA-MSDs with a mean 〈α〉=0.89. The width of the PDF (about ±0.2 around the mean) highlights, again, marked fluctuations between individual trajectories. In fact, similar fluctuations in the trajectory-wise values of α are expected already from mere statistical fluctuations due to fairly short trajectories (see [39] for discussion). Part of the width in p(α), however, may also reflect the spatially varying properties of the extract that is explored and reported on by different particles. Remarkably, trajectories of length N=70 and N=150 resulted in comparable PDFs and the same mean, i.e., longer trajectories were not biased toward lower scaling exponents. Notably, our observation of a subdiffusive motion of beads with 20 nm radius in Xenopus extracts is consistent with an earlier report [28] that reported scaling exponents in the range α∈[0.7,0.95], with lower values emerging for larger particles (radii in the range 0.1–1μm).

To further confirm and validate the significance of the mean exponent 〈α〉≈0.9, we exploited a bootstrapping approach [15]. Based on a total set of several hundred TA-MSDs, we randomly selected 100 trajectories and averaged these to a single, sub-ensemble averaged MSD from which we determined the scaling exponent α. This random drawing from the total set of TA-MSDs and the subsequent averaging was repeated 200 times to obtain a PDF for these scaling exponents. By construction, the width of this PDF can be expected to be much smaller [15], yielding a better estimate for the mean. Averaging over the subensemble (SE) of TA-MSDs was either done arithmetically (${\langle {\langle r^{2}\left(\tau \right)\rangle }_{t}\rangle }_{\mathrm{SE}}$), or by geometric averaging ($\exp[{\langle \log\left({\langle r^{2}\left(\tau \right)\rangle }_{t}\right)\rangle }_{\mathrm{SE}}]$). As a result, we found that the mean scaling exponent found by bootstrapping with arithmetic averaging was ${\langle \alpha \rangle }_{\mathrm{SE},a}=0.98$, which is consistent with normal diffusion. In contrast, geometric averaging yielded ${\langle \alpha \rangle }_{\mathrm{SE},g}=0.90$, in agreement with the mean of the raw PDF p(α) shown in Figure 2a. Since geometric averaging boils down to an arithmetic averaging of individual scaling exponents (due to the invoked logarithm), this result is, in fact, the more trustworthy approach for retrieving the average scaling exponent. Arithmetic averaging rather averages TA-MSDs with respect to their individual (and strongly fluctuating) diffusion coefficients, hence obscuring the mean scaling law and overestimating 〈α〉 (see [15] for another example). The difference in scaling obtained by the two averaging procedures may also be understood as a consequence of Jensen’s inequality. The mapping $\varphi :\alpha \mapsto t^{\alpha }$ is convex for any choice of a real number *t*. Jensen’s inequality then states that $t^{\langle \alpha \rangle }=\varphi \left(\langle \alpha \rangle \right)\leq \langle \varphi \left(\alpha \right)\rangle =\langle t^{\alpha }\rangle$, in line with our observation.

To check for the influence of localization errors when retrieving the scaling exponent from individual TA-MSDs, we took the following approach: using immobilized beads, we determined the contribution of the static localization offset to be a positive additive constant of about $8\times 10^{-4}\;\mu m^{2}$ (i.e., 20 nm accuracy of positions), whereas the dynamic localization error for our frame time and diffusion coefficients amounts to a negative constant with modulus $0.008\;\mu m^{2}$ or lower. Considering either of these two extreme values as additive constants to the power law Equation (2) while fitting TA-MSDs resulted in minor deviations of ±0.03 from the previously found value for 〈α〉. We therefore conclude that our SPT data show mild yet significant subdiffusion. In the following paragraphs, we further corroborate this conclusion by additional measures.

Let us now focus on the PDF of generalized diffusion coefficients, p(K), retrieved from individual TA-MSDs (Figure 2b). To remove the ambiguity of units for varying scaling exponents (units of *K* are $\mu m^{2}/s^{\alpha }$), we report these PDFs as a function of $K\times 1\;s^{\alpha }$, which represents the typical area covered by the particle within a second. As indicated already in the discussion of the previous paragraph, individual values of *K* varied considerably between trajectories, resulting in a very broad, almost lognormal-shaped PDF. Again, statistical fluctuations as well as locus-specific mobilities are likely to contribute to the width of p(K). In contrast to the anomaly exponents, a slight bias for smaller diffusion coefficients was visible for longer trajectories, seen as a slight shift of the peak in p(K) between N=70 and N=150 (cf. Figure 2b). Still, in both cases, the typical area explored within a second matches roughly with our calibration experiments in highly viscous glycerol–water mixtures (cf. Table 1), albeit the mean scaling exponent is clearly different. Furthermore, a scatter plot of trajectory-wise values for α and *K* (Figure 2c) highlights a correlation of these two quantities. This finding is in line with results of FBM simulations (M=2000 or M=1000 two-dimensional trajectories of length N=70 or N=150, obtained via the circulant method [40] with H=α/2=0.45 and $K=0.4\;\mu m^{2}/s^{\alpha }$; also shown in Figure 2c), which gives a first hint that an anti-persistent stochastic process underlies the acquired trajectories.

Let us briefly insert here an intuitive explanation of why a lognormal-like shape of p(K) is seen here and has also been reported frequently in the literature for other experiments (see [18,22,41,42,43,44,45,46,47,48] for examples). For the simplicity of the argument, we restrict ourselves to standard Brownian motion with Gaussian increment statistics and no memory kernel. Then, the diffusion constant $D=\langle {\langle r^{2}\left(\tau \right)\rangle }_{t}/\left(4\tau \right)\rangle$, as retrieved from a TA-MSD fit, is the finite mean of positive, squared Gaussian random numbers, $\Delta r^{2}$ (cf. Equation (1)). The associated PDF of $\vartheta =\Delta r^{2}/\langle \Delta r^{2}\rangle$ follows a special variant of the gamma distribution, known as Porter–Thomas distribution [49], $p\left(\vartheta \right)=\exp\left\{-\vartheta /2\right\}/\sqrt{2\pi \vartheta }$. Similar PDFs, featuring a power law that is cut by an exponential, have been observed, for example, for blinking quantum dots [50]. For power-law PDFs with a cutoff, it is known that a finite sum (or average) of random numbers will only slowly approach a Gaussian PDF, the hallmark of the central limit theorem. Indeed, numerically drawing *N* random numbers from the Porter–Thomas PDF and averaging (summing) them as $x=\sum_{i=1}^{N}\vartheta_{i}/N$ yields a PDF p(x) with a nonzero skewness $∼1/\sqrt{N}$ that resembles a lognormal PDF. Hence, even mere statistical reasons can lead already to an apparently lognormal PDF of diffusion constants if trajectories are short enough. Varying mobilities, encountered by particles in different spatial positions of a heterogeneous sample, broaden the PDF even further. Please also note that applying a logarithmic transformation is basically only one variant of the more general class of Box–Cox transformations $x\mapsto (x^{\lambda }-1)/\lambda$ [51], namely the one for λ=0. These transformations were introduced as a means to symmetrize a given data set, eventually yielding a Gaussian-shaped PDF of the transformed data when choosing the optimal λ. Restricting the choice to λ=0, i.e., simply logarithmizing the data, therefore will reduce the skewness in virtually all practical cases but may not yet completely symmetrize the data, leaving a residual non-zero skewness. As a consequence, any skewed data set will assume a more Gaussian shape upon applying a logarithmic transformation, although this mere statistical approach does, in general, not yet reveal the reason for the skewness and apparent lognormal PDF of the original data. In particular, inferring from an apparent lognormal shape of the PDF that the underlying data is the product of independent, identically-distributed variables may not be a compelling conjecture.

Coming back to our experimental data, we next explored whether the underlying random walk process is Gaussian. To this end, we considered the step increments $\left(\delta x_{i},\delta y_{i}\right)=\left(x_{i+n}-x_{i},y_{i+n}-y_{i}\right)$ taken within a period δt=nΔt. These follow a Gaussian PDF if the process is a simple FBM process. Recently, however, deviations from a Gaussian PDF were reported [14,15,52,53], highlighting heterogenous diffusion characteristics that could be rationalized by random walks with spatiotemporally fluctuating transport coefficients [54,55] and/or by systems with spatial disorders [56,57]. To account for the strongly fluctuating diffusion coefficients, we normalized the step increments δx and δy of each trajectory by the respective root-mean-square values. The resulting set of normalized steps did not exhibit significant differences between x- and y-coordinates. We therefore combined both into a single set of normalized increments, χ, which resulted in a symmetric PDF so that inspecting p(|χ|) was sufficient.

**Figure 3:**
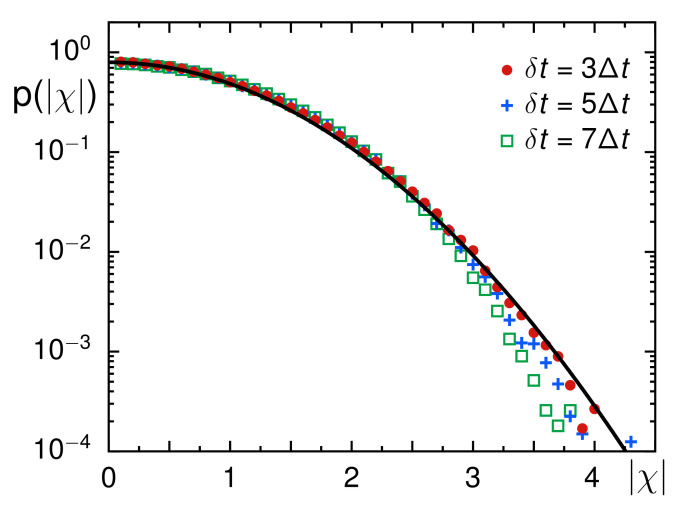
The PDF of normalized increments taken within a period δt, shown here as p(|χ|), complies well with a standard Gaussian (black full line) for different choices of δt (color-coded symbols). For δt≥5Δt and |χ|>3, consistently lower probabilities than the Gaussian benchmark are observed for unknown reasons.

Data for different δt show overall a very good agreement with a standard Gaussian and are incompatible with a simple exponential (Figure 3), indicating that no major diffusion heterogeneity is present in our SPT data from Xenopus extracts. For |χ|>3 and δt≥5Δt, the experimental PDF falls slightly below the Gaussian benchmark for unknown reasons. Despite this slight deviation, it appears fair to conclude that the trajectories emerged from a mildly subdiffusive Gaussian process, suggesting that FBM is the most likely model that describes our experimental data.

To follow up on this hypothesis and probe the existence of a non-trivial memory kernel in our experimental data, we employed the ensemble- and time-averaged velocity autocorrelation function (VACF) of each trajectory, defined as follows:

(4) $$C\left(\tau \right)={\langle \frac{{\langle v\left(t\right)v\left(t+\tau \right)\rangle }_{t}}{{\langle v{\left(t\right)}^{2}\rangle }_{t}}\rangle }_{E}\;\;,$$

with v(t)=[r(t+δt)−r(t)]/δt denoting the instantaneous velocity that is simply the two-dimensional step r(t+δt)−r(t) taken within integer multiples of the frame time, δt=nΔt.

It is convenient to rescale the lag time τ=kΔt with δt, yielding a dimensionless time ξ=τ/δt=k/n. For FBM, an analytical prediction for the VACF was derived [5,35]:

(5) $$C_{\mathrm{FBM}}\left(\xi \right)=\left\{{\left(\xi +1\right)}^{\alpha }+{\left|\xi -1\right|}^{\alpha }-2\xi^{\alpha }\right\}/2\;\;.$$

**Figure 4:**
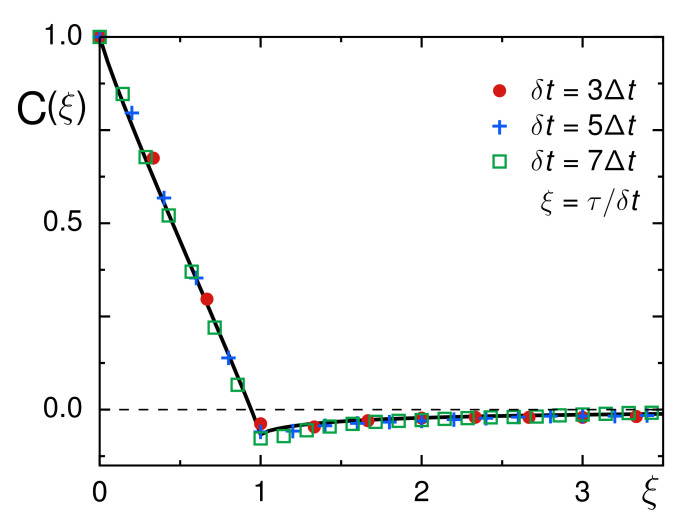
The normalized VACF, C(ξ), for different choices of δt (color-coded symbols) shows excellent agreement with the FBM prediction [Equation (5)] when inserting the mean scaling exponent 〈α〉=0.9 (full black line). In particular, a clearly negative value of C(ξ=1) confirms an antipersistent random walk, most likely of the FBM type. No significant changes of the VACF minimum are seen for different δt, confirming that trajectories are not plagued by localization errors.

The fact that the VACF does not depend on *n* and *k* but only on the ratio ξ=k/n reflects the self-similarity of FBM processes. Localization errors in SPT experiments can break this self-similarity [58,59], i.e., using different δt for rescaling τ to ξ leads to progressive deviations from Equation (5). In fact, very recently, the VACF was shown to be a sensitive reporter for detecting localization errors for FBM from the sub- to the superdiffusive regime [60], as even small localization errors lead to significant changes of C(ξ=1) at different choices of δt. In our case, however, rescaling with different δt lead to an almost perfect collapse of all data to the master curve predicted by Equation (5), see Figure 4. In fact, this finding is in favorable agreement with the earlier rheology results on Xenopus extracts that revealed a significant viscoelastic response [11,28], linking the anti-persistent dip in the VACF to a viscoelastic memory kernel of the medium. Moreover, the good agreement with Equation (5) for all choices of δt confirms our previous notion that localization offsets (to which VACFs are very sensitive) are negligible for our data.

As a further piece of evidence that the mild subdiffusion seen for particle motion in Xenopus extracts is due to an antipersistent FBM process, we probed the power-spectral density (PSD) of individual trajectories:

(6) $$S\left(f\right)=\frac{1}{T}{\left|\int_{0}^{T}e^{ift}x\left(t\right)dt\right|}^{2}+{\left|\int_{0}^{T}e^{ift}y\left(t\right)dt\right|}^{2}\;\;,$$

and the corresponding ensemble average, ${\langle S\left(f\right)\rangle }_{E}$. A wealth of analytical information is available for PSDs and their trajectory-wise fluctuations [61,62]. Alerted by the observations that arithmetic and geometric averaging can perturb power-law effects in the ensemble of trajectories (cf. above) we aimed at softening the influence of the grossly varying diffusion coefficients *K* in the subsequent analysis. Therefore, we normalized all trajectories by their respective root-mean-square step length within successive frames, in line with the approach taken when probing the Gaussian shape of the statistics of increments (cf. context of Figure 3).

**Figure 5:**
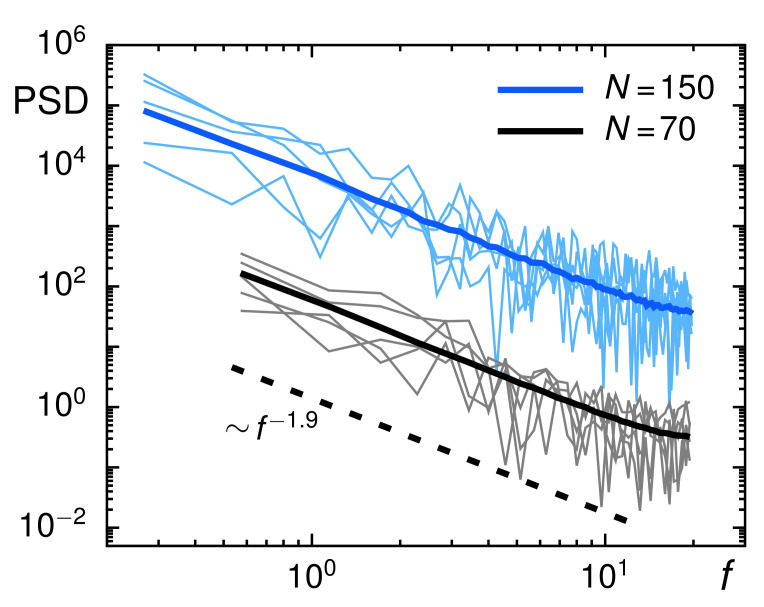
The PSD of individual trajectories (black and blue thin lines, representing trajectories with length N=70 and N=150, respectively) fluctuate around the ensemble-averaged PSD (thick colored lines). In both cases, the FBM prediction for a scaling $S\left(f\right)∼1/f^{1+\langle \alpha \rangle }$ (with 〈α〉=0.9, dashed line) are nicely met. For better visibility, data for N=150 have been shifted upward 100-fold.

**Figure 6:**
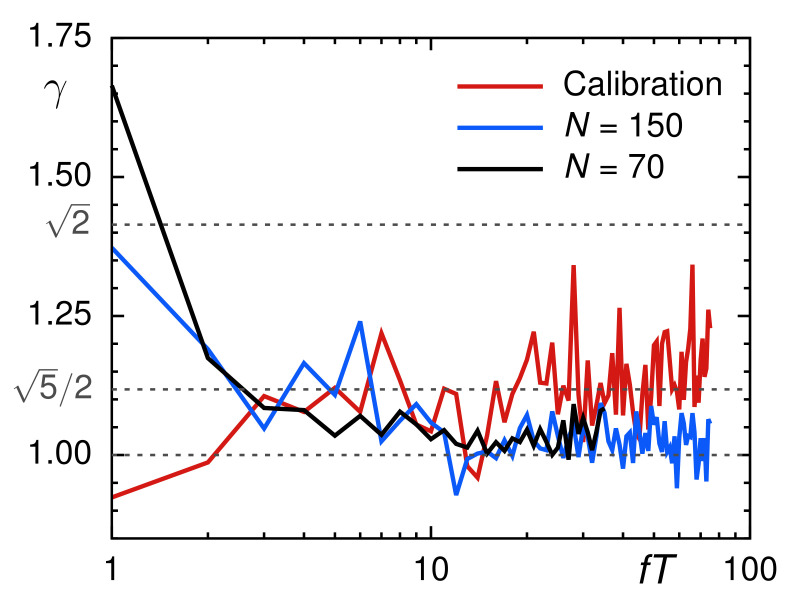
The coefficient of variation of individual PSDs with respect to the ensemble mean, γ(f), for normally diffusive trajectories from calibration experiments (red line) clearly assumes higher values than those for trajectories from the Xenopus extract (blue and black lines), irrespective of the trajectory length, *N*. As predicted for FBM, these subdiffusive SPT data converge toward γ=1, whereas normally diffusive data from calibration experiments converge to the predicted value $\gamma =\sqrt{5}/2$. Both are clearly distinct from the prediction for superdiffusive FBM motion, $\gamma =\sqrt{2}$. For convenience, frequencies *f* were made dimensionless by multiplication with the total time T=NΔt covered in each trajectory.

As expected, the ensemble-averaged PSD of these normalized trajectories (for N=70 and N=150) followed the analytical prediction $S\left(f\right)∼1/f^{1+\langle \alpha \rangle }$ around which PSDs of individual trajectories fluctuated to a considerable extension (Figure 5). These fluctuations encode another important hallmark of FBM via the coefficient of variation, defined as $\gamma \left(f\right)=\sigma /{\langle S\left(f\right)\rangle }_{E}$ with σ(f) denoting the standard deviation of trajectory-wise PSDs. For FBM, asymptotic values γ=1 for subdiffusion and $\gamma =\sqrt{5}/2$ for normal diffusion were predicted and verified before [61,62]. To calculate the coefficient of variation for our data, we randomly drew 1000 curves from the ensemble of one-dimensional TA-PSDs for the *x*- and *y*-direction and removed those 5% of TA-PSDs with the largest deviations from the ensemble-averaged PSD. The resulting values for γ fully complied with the FBM predictions (Figure 6). Normally diffusive trajectories from calibration experiments converge toward the predicted value $\gamma =\sqrt{5}/2$, whereas subdiffusive trajectories clearly assume lower values that eventually converge to the universal unity value for large frequencies.

Altogether, we conclude from the analyses of our SPT data that beads with 20 nm radius feature a mild antipersistent subdiffusion in Xenopus extract with a mean scaling exponent 〈α〉=0.9. Typical signatures of an FBM with a Hurst coefficient H=〈α〉/2=0.45 are clearly visible, suggesting that the viscoelasticity of the extract determines these distinct random-walk properties.

### 3.3. From Native to Pharmaceutically Treated Xenopus Extracts

**Figure 7:**
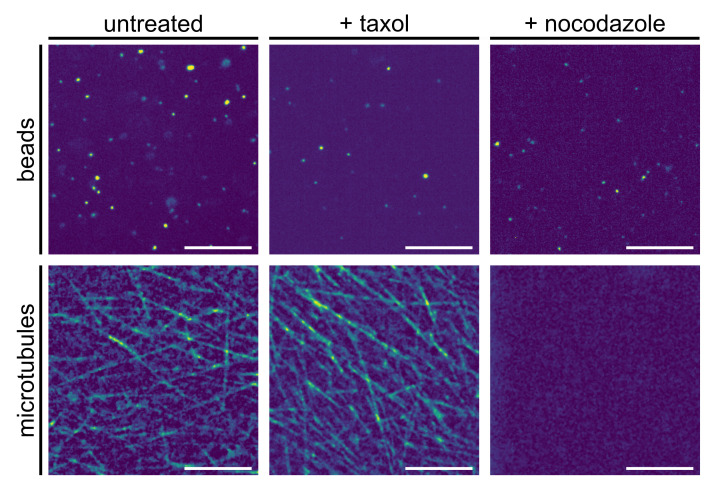
Representative fluorescence images of beads (upper panel) and microtubules (lower panel) in native and pharmaceutically treated Xenopus extracts (see Materials and Methods for details); scale bars indicate 10 μm. While native extracts feature a significant amount of microtubule filaments (left column), the addition of nocodazole completely eradicates these higher-order structures (right column). In contrast, stabilizing microtubules by taxol further enhances the ‘filament jungle’ (middle column).

To explore to what extent the observed subdiffusion is altered when challenging biochemical processes in the extract, we also performed single-particle tracking experiments after applying pharmaceuticals to the extract. In particular, we applied taxol or nocodazole to either stabilize or completely disrupt the microtubule filaments (see Materials and Methods for details). Typical fluorescence images of the extract (stained for the beads and the microtubule filaments) highlight the strong differences between untreated and chemically challenged extracts (Figure 7). While untreated extracts feature some microtubule filaments that might obstruct the free diffusion of beads, the addition of nocodazole completely erradicates these structures, potentially resulting in a decreased obstruction of bead motion. In contrast, the addition of taxol stabilizes microtubules and, therefore, even enhances the gel-like geometry within the extract.

Somewhat unexpectedly, however, altering the microtubule filament array had, on average, only minor effects (Table 2). While disrupting microtubules had, on average, no significant effect at all (with 〈α〉 and 〈K〉 being almost unchanged), the addition of taxol induced a slight enhancement of the subdiffusion, i.e., a lower 〈α〉, in line with the notion that increased density of filaments may further hamper the beads’ free diffusion. Still, the effect is fairly small when bearing in mind that scaling exponents around and below α≈0.5 were reported already for similar sized particles in the comparable cytoplasm of living cells [6,11,15]. Our findings, therefore, indicate that mainly macromolecular crowding of the fluid on length scales ≪1μm, which induces a viscoelastic memory kernel, underlies the observed subdiffusion. Higher-order structures, such as cytoskeletal assemblies or endomembranes, appear to be less important for the observed (sub)diffusion of beads in Xenopus extract.

To complement these insights, we also applied non-hydrolizable analogues of ATP and GTP to prevent non-equilibrium processes that are fueled by these nucleotides (see Materials and Methods for details). In this case, the extract became very heterogeneous with tracking results from separated loci differing strongly (from total immobilization up to normal diffusion). Removing the immobilized tracks, the average behavior was in reasonable agreement with our findings for untreated and taxol-treated extracts. This finding suggests that (apart from immobilization loci) the ATP- and GTP-dependent processes are also of little importance for the motion of small beads. As a caveat, we would like to emphasize, however, that all of these findings might be subject to change when larger particles or different surface properties are considered, as these might interact differently with macromolecules and higher-order structures. In support of this statement, we would like to refer the reader to previous measurements on the diffusion of quantum dots in the cytoplasm of mammalian cells, where strong variations in the mobility and apparent diffusion anomaly were observed upon varying the particles’ surface chemistry [63]. In fact, understanding anomalous diffusion in complex media at non-equilibrium conditions, e.g., in the crowded interior of living cells, is still a major challenge (see [64] for a recent study).

## 4. Conclusions

In summary, we have shown here that beads with 20 nm radius explore cell extracts from eggs of *Xenopus laevis* by mild subdiffusion that bears all properties of a FBM random walk. This mode of motion is largely conserved when treating the extract with pharmaceuticals that alter microtubule filaments or ATP/GTP-dependent processes. Therefore, the emergence of subdiffusion is most likely a consequence of the extracts’ viscoelasticity that is induced by a high degree of macromolecular crowding, albeit changes in the beads’ surface chemistry might also enhance or lower the diffusion anomaly due to transient and unspecific interactions with larger structures in the fluid [63]. In any case, given that even mild subdiffusion was predicted and observed to significantly alter biochemical reactions (see, e.g., [65,66,67]) our data provide a helpful clue for a deeper understanding of self-organization and pattern formation processes in cell extracts.

## Figures and Tables

**Table 1:** Summary of glycerol concentrations (weight percent) in glycerol–water mixtures, along with the respective viscosities η, and predicted diffusion constants *D*. Average scaling exponents 〈α〉 (found via fitting all TA-MSDs for trajectories of 20 nm radius particles as described in the main text, followed by averaging the individual values of α) are near to unity and mean diffusion coefficients 〈K〉 (also obtained by averaging the results for individual TA-MSDs) compare favorably to the predicted values of *D*. Result for trajectories with length N=70 and N=150 are given in upper and lower lines, respectively. The ensemble size of evaluated trajectories for the respective condition is given by *M*.

glyc.	η [Pas]	*D* [$\mu m^{2}/s$]	〈α〉	〈K〉 [$\mu m^{2}/s^{\alpha }$]	*M*
70%	0.016	0.67	1.00	0.56	929
			0.99	0.45	67
75%	0.023	0.46	1.01	0.42	1513
			1.03	0.35	185
80%	0.035	0.30	1.01	0.28	2158
			1.03	0.24	250
85%	0.055	0.19	1.01	0.20	4426
			1.02	0.18	749
90%	0.096	0.11	1.00	0.13	4668
			1.02	0.12	980

**Table 2:** Summary of results found for different conditions of Xenopus extracts. Data for trajectories with length N=70 and N=150 are given in upper and lower lines, respectively. The ensemble size for the respective condition is given by *M*.

cond.	〈α〉	$\langle K\rangle \;\left[\mu m^{2}/s^{\alpha }\right]$	*M*
untreated	0.89	0.39	3085
	0.88	0.27	582
+taxol	0.83	0.33	2548
	0.81	0.25	606
+nocodazol	0.89	0.37	1126
	0.81	0.17	130
+ATPγS +GTPγS	0.85	0.31	915
	0.81	0.10	189
+ATPγS +GTPγS +noc.	0.88	0.35	2646
	0.83	0.23	471

## Data Availability

The data presented in this study are available from the corresponding author upon reasonable request.
